# Elucidation of Ubiquitin-Related Functions via an Ubiquitin Overexpression Approach

**DOI:** 10.3390/cells13232011

**Published:** 2024-12-05

**Authors:** Ryo Masuda, Munetaka Yoshikawa, Ryota Moriuchi, Yumiko Oba, Hideo Dohra, Yoko Kimura

**Affiliations:** 1 Graduate School of Integrated Science and Technology, Shizuoka University, Shizuoka 422-8529, Japandora.hideo@shizuoka.ac.jp (H.D.); 2 Shizuoka Instrumental Analysis Center, Shizuoka University, Shizuoka 422-8529, Japan; moriuchi.ryota@shizuoka.ac.jp; 3 Department of Agriculture, Shizuoka University, Shizuoka 422-8529, Japan; oba.yumiko.18@shizuoka.ac.jp; 4 Research Institute of Green Science and Technology, Shizuoka University, Shizuoka 422-8529, Japan

**Keywords:** ubiquitin, Spc2, signal peptidase, Hrd1, yeast

## Abstract

To identify new ubiquitin-related functions using yeast, we searched for mutants conferring a temperature-sensitivity phenotype that could be rescued through ubiquitin overexpression. Screening of mutants using this overexpression strategy identified *SPC2*, which encodes a subunit of the endoplasmic reticulum (ER) signal peptidase complex (SPC). Ubiquitin overexpression rescued a high-temperature sensitivity of *spc2* deletion mutant, suggesting that ubiquitin could compensate for Spc2 loss-of-function at high temperatures. The double mutant of Spc2 and Hrd1, an ER E3 ubiquitin ligase, showed a synergistic growth defect at higher temperatures. A weak genetic interaction was also observed between spc2Δ and *cdc48-3* mutation. The results suggest a close functional relationship between SPC and the ubiquitin–proteasome system in yeast and further provide proof-of-principle for this ubiquitin overexpression approach to identify novel ubiquitin-related genes and associated cellular processes.

## 1. Introduction

Ubiquitin is a small, highly conserved, 76-amino-acid protein that modulates the fate and function of numerous target proteins by reversible, enzyme-catalyzed covalent attachment to lysine residues, termed ubiquitination [1,2,3]. Cellular functions reversibly regulated via ubiquitination include protein degradation, protein trafficking, cell-cycle progression, genome surveillance, apoptosis, and signal transduction. Moreover, ubiquitination functions in the quality control of organelles such as the endoplasmic reticulum (ER) and mitochondria. For example, ubiquitin contributes to the maintenance of ER function through the ER-associated protein degradation (ERAD) system, ER stress-induced pre-emptive quality control (ERpQC) system, and the recently identified ER membrane protein quality control system mediated by the signal peptidase complex (SPC) [4,5,6,7].

Reversible ubiquitination is catalyzed by the sequential actions of three enzyme classes [1,8]: ubiquitin-activating (E1) enzymes, ubiquitin-conjugating (E2) enzymes, and ubiquitin ligase (E3) enzymes. The best-described function of ubiquitination is the tagging of target proteins for selective proteolysis by the proteasome. Here, several ubiquitins are covalently added to a substrate successively, producing a substrate conjugated with a chain of ubiquitins. The ubiquitinated substrates are recognized and degraded by proteasomes after the polyubiquitin chain is cut off by deubiquitinating enzymes. There is mounting evidence, however, that ubiquitination is also involved in numerous additional processes [9,10,11,12]. For example, ubiquitination is critical for vacuolar sorting of both endocytic and biosynthetic membrane proteins. At the plasma membrane, ubiquitin serves as a signal for endocytosis, while at the endosome, it serves as a signal to sort cargo proteins into multivesicular bodies, which then fuse with lysosomes/vacuoles for degradation.

In eukaryotes, ubiquitin is initially expressed in the form of different precursors: polyubiquitin, a linear chain consisting of four or more ubiquitin copies in a head-to-tail configuration, and in fusion proteins between ubiquitin such as Ub_L40_ and Ub_S27_ containing the large and small ribosomal polypeptides, L40 and S27, respectively [3]. These ubiquitin precursors are cleaved by deubiquitinating enzymes to release identical functional monomeric ubiquitin units. In yeast, which has four ubiquitin-coding genes, the single polyubiquitin gene *UBI4* is not required under vegetative conditions; however, deletion mutant (*ubi4*Δ) cells show a growth defect under chronic exposure to sublethal high temperatures due to ubiquitin deficiency [13]. Several mutants harboring disrupted genes involved in the ubiquitin–proteasome system (UPS) have been identified that also demonstrate growth defects when exposed to various stress conditions, such as heat stress, canavanine, and cadmium-induced ER stress [13,14,15]. It has been asserted that the intact ubiquitin system is necessary to remove misfolded proteins, which are induced by stresses. Thus, these mutants would not be able to cope with these proteins due to the defects in the degradation systems, resulting in the impairment of cell growth. In addition, some of these growth-impaired phenotypes were rescued through ubiquitin overexpression [16], likely because ubiquitin overexpression overcomes insufficient endogenous ubiquitination reactions or supplying ubiquitin if a ubiquitin shortage occurs, thereby sustaining growth-related processes.

Based on these previous reports, we reasoned that proteins with previously unknown ubiquitin-related functions in yeast could be identified by screening mutants for ubiquitin-mediated rescue of heat-sensitive growth impairment. Therefore, we generated mutants with temperature-sensitive phenotypes using ultraviolet irradiation and screened for those with heat-induced growth impairment reversed through ubiquitin overexpression. Using this strategy, we identified *SPC2,* which encodes a subunit of SPC in the ER as a ubiquitin-related gene.

## 2. Materials and Methods

### 2.1. Yeast Strains

Yeast strains W303 (MATa *ade2-1 can1-100 his3-12,16 leu2-3,112 trp1-1 ura3-1*) and BY4741 (MATa *his3*Δ*1 leu2*Δ*0 met150 ura3*Δ*0*) were used as the wild types (WTs) in the present study. The mutant strains used in this study are listed in Supplementary Table S1. The *SPC2* deletion strain of W303 background was constructed by transforming W303 cells with the *spc2::KanMX* cassette amplified from the BY4741 *spc2*Δ strain using PCR and the primers 1454 and 1455 (Table S2). The *spc2*Δ*hrd1*Δ and *spc2*Δ*hrd1*Δ mutants were created as follows: first, *spc2*Δ*:KanMX* in Y1479 was changed to *spc2*Δ*::hpnNT1* to make Y2204; and a PCR fragment of *hrd1*Δ*::KanMX* or *doa10*Δ*::KanMX* DNA was transformed toY2204.

### 2.2. Media

**Figure 1:**
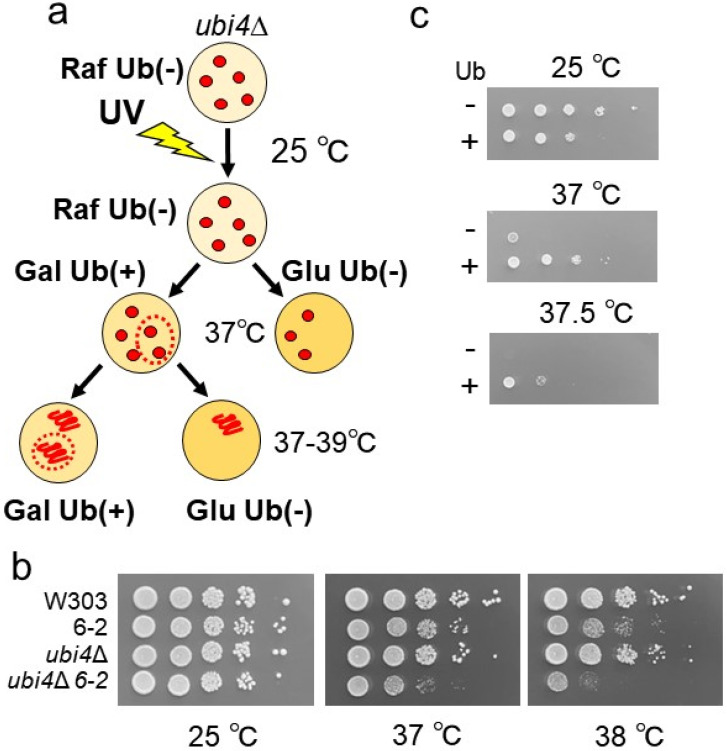
Isolation of the mutants with a temperature-sensitive phenotype that were rescued through ubiquitin overexpression. (**a**) Schematic flow of screening of the mutants. The *ubi4*Δ cells (Y914) with a plasmid expressing ubiquitin under a galactose-inducible promoter were mutagenized with UV. In this screening, on galactose but not on raffinose or glucose media, overexpression of ubiquitin was induced. Temperature-sensitive mutants were screened on the growth on glucose media, and for suppressing the temperature-sensitivity on galactose at 37–39 °C. (**b**) Growth of WT(W303), 6-2 *UBI4* (Y1355), *ubi4*Δ (Y914), and 6-2 *ubi4*Δ (Y2198) mutants at different temperatures. Cells were serially diluted 10-fold, spotted on YPAD, and grown at the indicated temperatures for 3 days. (**c**) 6-2 *ubi4*Δ mutants (Y2198) with a vector or a plasmid expressing ubiquitin under a galactose-inducible promoter (E889) were grown in SRaf−Leu media, serially diluted by 10-fold, spotted on SGal/Raf−Leu plates and grown at the indicated temperatures for 5 days.

Yeast cells were grown in YPAD medium (1% yeast extract, 2% bactopeptone, 2% glucose, and 0.004% adenine), synthetic medium (SD; 0.67% yeast nitrogen base and 2% glucose, raffinose or galactose, supplemented with amino acids), or synthetic casamino medium (SC; 0.67% yeast nitrogen base, 2% glucose, 0.5% casamino acids, and, if necessary, supplementation of tryptophan, uracil, or adenine as indicated). For the spotting assay in Figure 1c, raffinose was added at the final concentration of 2% in addition to 2% galactose because yeast growth is very slow in galactose media.

### 2.3. Plasmids

The plasmids used in this study are listed in Supplementary Table S3. The plasmid pRS315-GAL1p-Ub (E889) encoding ubiquitin under control of the *GAL1* promoter was constructed by ligating SacI-HindIII pRS315, the HindIII-BamHI fragment of plasmid M212 containing the *GAL1* promoter region, and the BamHI-SacI region of plasmid E864 containing the fifth ubiquitin-coding region of *UBI4* plus 200 bp downstream of *UBI4*. The plasmid pRS316-SPC2 (E974) was created as follows: The *SPC2* gene was cloned from genomic DNA by PCR using primers 1386 and 1387 (Table S3). Then, the PCR product was cut with KpnI and SacI and ligated with the KpnI-SacI fragment of pRS316. The plasmid,= pRS316-HRD1 was created by ligating the EcoR1- XhoI fragment from plasmid pKN303 (a gift from Dr. K. Nakatsukasa, Nagoya City Univ. Japan), with the EcoR1-XhoI from pRS316.

### 2.4. UV Mutagenesis

The deletion mutant *ubi4*Δ (Y914) was transformed with a plasmid expressing ubiquitin under control of the *GAL1* promoter (E889). Transformed cells were grown to log phase, pelleted, washed with distilled water, and plated at approximately 1000 cells/plate on S Raffinose–Leu medium. Subsequently, the plates were exposed to UV radiation at doses ranging from 1000 to 9000 μJ/cm^2^ and incubated at 25 °C. When colonies were very small, replicas were transferred to S Galactose–Leu and SD–Leu plates and incubated at 37 °C for 3 days. Cells that continued to grow on S Galactose–Leu but not on SD–Leu were isolated and retested for growth on the same media.

### 2.5. Genome Resequencing and Genome-Wide SNP Calling

Genomic DNA was extracted from yeast cells using the following protocol: Cells were pelleted, resuspended in 12 mL of spheroplasting buffer (0.9 M sorbitol, 50 mM EDTA [pH 8], 50 mM DTT) plus 0.75 mg zymolyase 100T, and incubated at 30 °C for 30 min. A 500 μL volume of 1.5 mg/mL zymolyase 100T solution was added to the suspension, followed by another 30 min incubation at 30 °C. Spheloplasted cells were then pelleted, washed, and resuspended in 5 mL of 50 mM EDTA plus 0.5 mL of 10% SDS. This mixture was heated to 65 °C for 30 min, followed by the addition of 1.7 mL of 5 M potassium acetate. This new mixture was placed on ice for 60 min and centrifuged at 10,000 rpm for 30 min at 4 °C. The supernatant (~4.5 mL) was mixed with an equal volume of ethanol and centrifuged. The pellet (raw genomic DNA) was washed successively with 70% and 50% ethanol, dried, suspended in 1 mL TE buffer, and incubated for 10 min at 42 °C. This mixture was centrifuged, and the supernatant incubated with 15 mL of 10 mg/mL RNase for 30 min at 37 °C. The remaining DNA was reprecipitated by addition of 1 mL isopropanol, and the pellet was washed with 50% isopropanol. If necessary, RNase treatment and isopropanol extraction steps were repeated. Genomic DNA was further purified using spin columns followed by proteinase K treatment and repurification using DNA spin columns. Genomic DNA was then fragmented using the Covaris acoustic solubilizer.

Paired-end libraries prepared by the TruSeq DNA Nano Kit (Illumina, San Diego, CA, USA) were sequenced using the MiSeq platform (Illumina) (301-bp paired-end) at the Functional Genomics Section, Shizuoka Instrumental Analysis Center, Shizuoka University (Supplementary Table S4). The raw reads were cleaned up using Trimmomatic ver. 0.39 [17] by removing low-quality ends (quality score, <15), adapter sequences, the last bases (301st), and reads less than 35 bp. SNP distribution was analyzed based on the variant calling pipeline using GATK4 (https://gencore.bio.nyu.edu/variant-calling-pipeline-gatk4/, accessed on 6 January 2024). Summarily, the cleaned reads were mapped to the *Saccharomyces cerevisiae* W303 reference genome (GCA_002163515.1) using BWA-MEM ver. 0.7.17 [18], duplicates were marked and sorted with Picard ver. 3.1.1 (https://broadinstitute.github.io/picard/, accessed on 5 January 2024), and variant SNPs and indels were identified with GATK ver. 4.5.0. [19]. The annotation of genetic variants and the prediction of their effects were performed using SnpEff ver. 5.2 [20]. Identified SNPs and indels were confirmed by visualizing the aligned reads using the Integrative Genomics Viewer [21].

The raw read sequences used in this study have been deposited in the DDBJ Sequence Read Archive (DRA) under the accession numbers DRR550211 (strain W303) and DRR550212 (mutant strain).

### 2.6. Immunoblot Analysis

Immunoblot analysis of whole-cell extracts was performed as described previously [22]. Western blot membranes were incubated with anti-Kar2 antibody (a gift from Dr. Kenji Kohno, NAIST, Japan) or anti-yeast phosphoglycerate kinase monoclonal antibody (Thermo Fisher, Waltham, MA, USA), followed by horseradish peroxidase (HRP)-conjugated anti-rabbit IgG (#NA934V; Cytiva, Waltham, MA, USA) or anti-mouse IgG (#NA931V; Cytiva, Waltham, MA, USA), respectively. Target bands were visualized using a chemiluminescent reagent. For the detection of ubiquitin, HRP-conjugated P4D1 mouse monoclonal antibody (Santa Cruz Technology, Dallas, TX, USA) was used.

## 3. Results

### 3.1. Identification of the spc2 Mutation

To identify novel ubiquitin-related functions, we generated temperature-sensitive mutants with growth defects reversible via ubiquitin overexpression (Figure 1a). Briefly, *ubi4*Δ (*ubi4*Δ::KanMX) cells, which are sensitized to heat stress due to an endogenous ubiquitin deficiency, were transformed with a plasmid expressing ubiquitin under control of the *GAL1* promoter. The plasmid-harboring cells were mutagenized through UV exposure, and plated on raffinose media at 25 °C. Once colonies appeared, plates were replicated on both glucose and galactose media and incubated at 37 °C. In galactose media where *GAL1* promoter is turned on, ubiquitin is overproduced from the plasmid; whereas in glucose media where *GAL1* promoter is repressed, ubiquitin expression from the plasmid is repressed, creating matched populations of ubiquitin-overexpressing and ubiquitin-deficient clones. Screening of about 30,000 colonies identified three mutants that grew on galactose plates but not on glucose plates at 37 °C, or that grew a little bit (if at all) on a glucose plate at 37 °C (i.e., with a temperature-sensitive growth deficiency). These mutants were crossed with WT cells, and the obtained diploid cells were sporulated to make tetrads. Tetrads were then dissected, and segregates were analyzed for temperature-sensitivity and G418 resistance. One mutant showed 2:2-separated temperature-sensitive growth regardless of the presence of the *ubi4*Δ mutation. We designated this mutation as 6-2 and observed that the temperature-sensitivity conferred by 6-2 alone was weaker than that of the mutant harboring both 6-2 and *ubi4*Δ (Figure 1b). We confirmed that the temperature-sensitive growth of 6-2 *ubi4*Δ mutant was suppressed through ubiquitin over-expression (Figure 1c). These results suggest that a UV-induced mutation in a single gene acted in conjunction with *ubi4*Δ to confer the highly temperature-sensitive phenotype.

**Figure 2:**
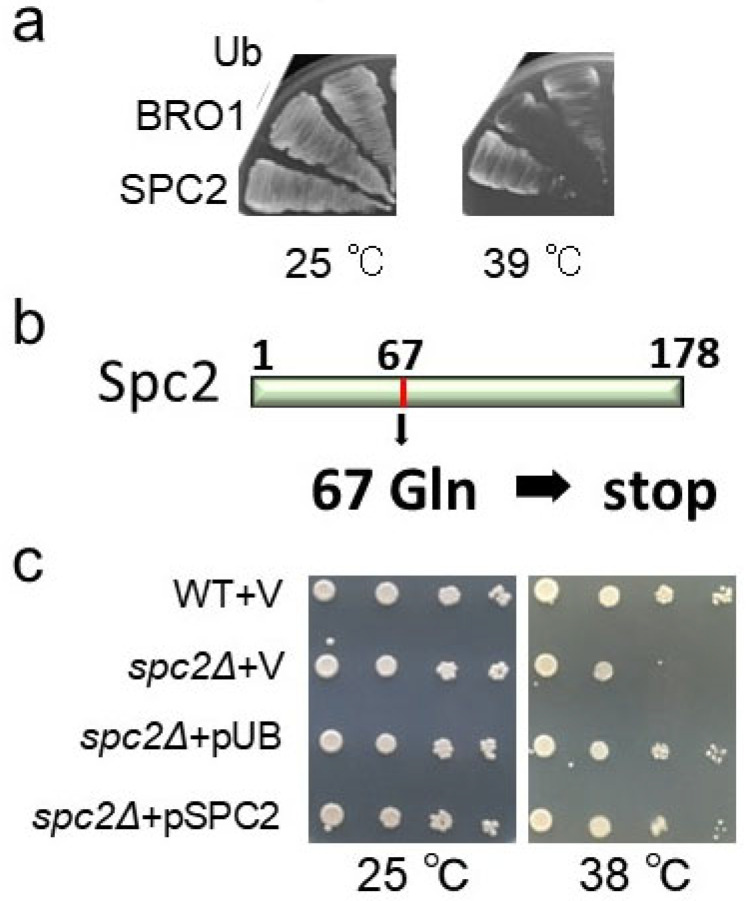
Characterization of the *spc2* mutation: (**a**) suppression of the temperature-sensitive growth deficit of 6-2 mutant (Y1355) via the introduction of a plasmid with *SPC2* DNA and a plasmid overexpressing ubiquitin under constitutive *GAP* promoter(E446), but not a plasmid with *BRO1* DNA; (**b**) predicted amino acid change of Spc2 by the *spc2* mutation; (**c**) suppression of the temperature-sensitive growth deficit in *spc2*Δ mutant via the overexpression of ubiquitin. Wild-type (BY4741) cells transformed with an empty vector (pRS316) and *spc2*Δ cells (Y1479) transformed with vector (pRS316), a plasmid expressing ubiquitin under constitutive GAP1 promoter (pUB, E446), or Spc2 expressing plasmid (E974), were serially diluted 10-fold, spotted on SC–ura plates, and grown at the indicated temperatures for 3 days.

Using the 6-2 mutant which was backcrossed with wild-type cells three times, genome resequencing and genome-wide SNP calling were performed. These analyses revealed nucleotide changes in two genes: *SPC2*, encoding signal peptidase complex subunit 2, and *BRO1*, encoding a vacuolar sorting protein in the mutant (Table 1). To test which of these genes is responsible for the temperature-sensitive phenotype, plasmids containing each gene were introduced separately into the mutant. The plasmid containing *SPC2*, but not *BRO1* DNA, rescued the temperature-sensitive phenotype of the mutant, suggesting that the observed phenotype is conferred by the *spc2* mutation (Figure 2a).

### 3.2. Characterization of the spc2 Mutation

The *SPC2* gene encodes a 178-amino acid subunit of the SPC localized within the ER. In yeast, the SPC consists of four subunits [23]: Sec11, Spc3, Spc1, and Spc2. Of these, Sec11 and Spc3 are essential for cleavage of the substrate, whereas Spc1 and Spc2 are accessory proteins for the enzyme [24]. In the 6-2 mutant, a nucleotide change from C to T was detected at position 199, which is predicted to encode a 66-amino acid truncated Spc2 protein (Table 1, Figure 2b).

This temperature-sensitive growth defect was lost in heterozygotes of the mutant × WT cross, indicating that the mutation was recessive. Therefore, we next examined the phenotype of the *spc2*Δ deletion mutant in response to temperature stress and potential rescue by ubiquitin (Figure 2c). The mutant showed a modest growth defect at higher temperatures that was moderately greater on the BY4741 strain background than the W303 strain background. We found that temperature-sensitive phenotype of *spc2*Δ was suppressed by overexpression of ubiquitin as well as the introduction of plasmid expressing Spc2. These results suggested that ubiquitin overexpression covered the deficiency of Spc2 at higher temperatures.

### 3.3. Genetic Interaction of spc2Δ with hrd1Δ

**Figure 3:**
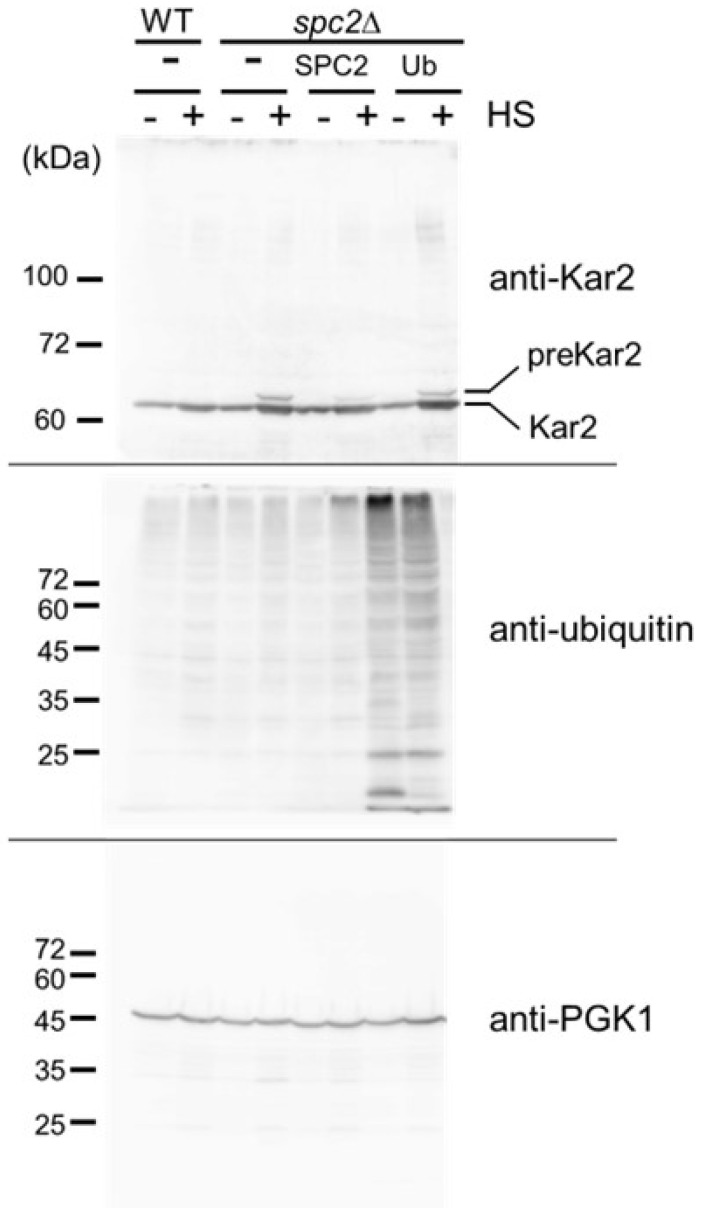
PreKar2 accumulation. WT(BY4741) cells with a vector and *spc2*Δ cells with a vector (pRS316), SPC2-expresing plasmid (E974), or ubiquitin overexpressing plasmid (E446), were grown at 25 °C and 40.5 °C for 3 h. Blots were separately prepared and reacted with anti-Kar2, anti-ubiquitin, and anti-PGK1 antibody. PGK1 blot is a control for protein loading.

A ERpQC system has been described in mammalian cells that functions in the removal of precursor polypeptides with a signal peptide and ensuing degradation by the UPS before ER entry when ER translocation is attenuated by ER stress [4]. Mullins and colleagues (1996) reported that signal peptidase activity remained unaffected by *spc2*Δ deletion at normal temperatures. However, they showed that at 42 °C for 3 h, preprotein processing becomes defective, leading to the accumulation of precursor proteins such as preKar2p and procarboxypeptidase. Therefore, we hypothesized that an ERpQC-like system may operate at high temperatures in yeast and tested the possibility that ubiquitin overexpression reduces accumulation of preKar2p at high temperatures. Substantial preKar2p accumulation was detected in the lysate from *spc2*Δ cells but not from WT cells grown at 40.5 °C for 3 h, according to Western blotting using an anti-Kar2 antibody that detects both preKar2p and mature Kar2p (Figure 3) [25,26]. Through the expression of Spc2 into the *spc2*Δ mutant, preKar2p accumulation was reduced. However, overexpression of ubiquitin did not reduce the accumulation of preKar2p significantly under this experimental condition.

**Figure 4:**
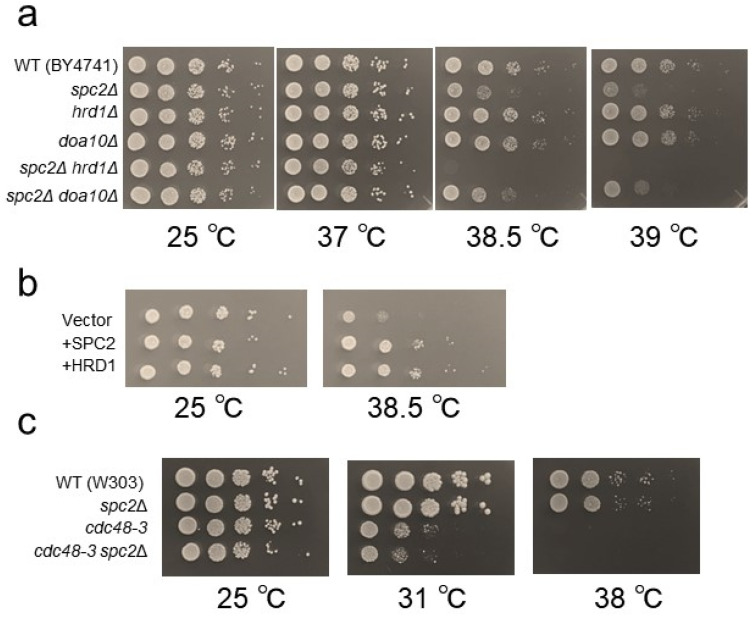
Genetic interaction between *hrd1*Δ and *spc2*Δ or *cdc48-3*. (**a**) Indicated cells of BY4741 strains were diluted serially 10-fold, spotted on YPAD plates, and grown at the indicated temperatures at 25 °C for two days and at 37 °C, 38.5 °C, and 39 °C for 3 days. WT(BY4741), *spc2*Δ (Y1479), *hrd1*Δ(Y1533), *doa10*Δ(Y1532), *spc2*Δ*hrd1*Δ (Y2211), and *spc2*Δ*doa10*Δ (Y2212) were used. (**b**) Suppression of temperature-sensitive phenotype of *spc2*Δ*hrd1*Δ mutant by introduction of a plasmid expressing Spc2 or Hrd1. Cells were spotted on SC−Ura and grown at the indicated temperatures for 2 days. (**c**) Growth of WT(W303), *spc2*Δ(Y1355), *cdc48-3* (Y204), and *cdc48-3 spc2*Δ (Y2198) mutants at different temperatures. Cells were serially diluted 10-fold, spotted on YPD, and grown at 25 °C for two days and at 31 °C and 38 °C for 3 days.

In addition to the well-understood functions of SPC for removing signal peptides from proteins entering the ER, Zanotti et al. (2022) revealed another important function of SPC in the quality control of ER membrane proteins [5]. Specifically, they showed that the SPC post-translationally cleaves ER membrane proteins that fail to fold correctly or assemble in the ER. This process involves Hrd1, an E3 ligase known to function in ERAD, which targets substrates for degradation in the UPS. In ERAD, it has been shown that as well as functioning as an E3 ligase, Hrd1 contributes to the retro-translocation of polypeptides from the ER lumen and membrane to the cytosol [27]. Further, it has been shown that Hrd1 and SPC interact physically in mammalian cells and that Hrd1 participates in the clearance of SPC-generated fragments [5]. Also, Hrd1 is implicated in the ERpQC system [4]. Therefore, we investigated the potential genetic interaction between *spc2*Δ and *hrd1*Δ mutations and found a synergistic growth defect in the *spc2*Δ*hrd1*Δ double mutant at high temperatures compared to individual mutants (Figure 4a). The temperature-sensitive growth of the *spc2*Δ*hrd1*Δ double mutant was rescued by the expression of Hrd1 and Spc2 (Figure 4b). No genetic interaction was detected in a strain harboring the *spc2*Δ mutant and a deletion mutation of the ER ubiquitin ligase Doa10.

Moreover, we observed a weak synergistic growth defect at 31 °C in a double mutant of *spc2*Δ and *cdc48-3,* a temperature-sensitive allele of *CDC48* which is essential for yeast (Figure 4c). The Cdc48 protein, a mammalian homolog of p97/VCP, is a ubiquitin-specific chaperone that unfolds ubiquitinated substrates before degradation by the proteasome and also functions in ERAD, in which Cdc48 pulls out the protein from the ER membrane [28,29,30,31]. Collectively, these results suggest a relationship among Spc2, ubiquitin, Hrd1, and Cdc48 in yeast.

## 4. Discussion

Utilizing a combined UV mutagenesis and ubiquitin-mediated phenotype rescue screening strategy, we present evidence that the SPC subunit Spc2 is important for a novel ubiquitin-related function and for UPS activity in yeast. We identified a *spc2* loss-of-function mutant (*spc2*Δ) with a temperature-sensitive growth deficit that was rescued via ubiquitin overexpression, suggesting that ubiquitin may preserve or replace Spc2 growth-related functions at high temperatures. In addition, we revealed genetic interactions of Spc2 with the UPS components Hrd1 and Cdc48.

The contributions of Spc2 to the UPS are still unknown in yeast. The *spc2*Δ mutant exhibited accumulation of Kar2 proprotein that was not reversed through ubiquitin overexpression, suggesting that the mechanism for phenotype rescue may be independent of proprotein removal from the ER via ubiquitination. Nonetheless, analysis of additional ER lumen proteins is required for confirmation. Elucidating the relationship between Spc2 and the UPS in yeast may require identification of specific substrate(s), possibly ER membrane proteins. Like mammalian cells, yeast may express hidden or cryptic substrates of signal peptidase that are removed by the UPS [5]. We speculate that such substrates increase at higher temperatures.

In conclusion, using a ubiquitin overexpression approach, we identified Spc2 as a novel ubiquitin- and UPS-related protein. This ubiquitin overexpression approach could be useful for identifying additional ubiquitin-related genes and cellular processes.

## Figures and Tables

**Table 1:** Details of the SNPs detected in the mutant with a temperature-sensitive phenotype.

	Position	Reference	Variation	Quality	Coverage ^a^	Impact	Functional Class	Gene	DNA Changes	Protein Changes
XIII	159,529	C	T	889.48	42	HIGH	stop_gained	*SPC2*	c.199C>T	p.Gln67 *
XVI	566,232	C	T	846.48	47	HIGH	stop_gained	*BRO1*	c.1342C>T	p.Gln448 *

^a^ Filtered depth, at the sample level. * Translation stop here.

## Data Availability

The sequencing data used in this study have been deposited in the GenBank/EMBL/DDBJ under the accession numbers PRJDB17962.

## Supplementary Materials

The following supporting information can be downloaded at https://www.mdpi.com/article/10.3390/cells13232011/s1: Table S1: Strains used in this study; Table S2: Oligonucleotide primers used in this study; Table S3: Plasmids used in this study; Table S4: Information of genome sequencing in this study. References [32,33,34,35] are cited in Supplementary Materials.
